# Potential metabolomic biomarkers for the identification and diagnosis of type A acute aortic dissection in patients with hypertension

**DOI:** 10.3389/fcvm.2022.1019598

**Published:** 2022-11-07

**Authors:** Xiao-Bin Hao, Yue Han, Er-Ru Ni, Ming-Cai Ye, Gang Li, Xi-Jie Wu, Hai-Feng Qiang, Jun Zhao

**Affiliations:** ^1^Department of Thoracic Surgery, The First Affiliated Hospital of Soochow University, Medical College of Soochow University, Suzhou, China; ^2^Xiamen Cardiovascular Hospital of Xiamen University, School of Medicine, Xiamen University, Xiamen, China

**Keywords:** acute aortic dissection, hypertension, metabolites, lipid metabolism, dimethylglycine, hydrocortisone

## Abstract

**Objectives:**

Most patients with acute aortic dissection (AAD) have a history of hypertension. Diagnosis of AAD in patients with hypertension at an early stage is complicated and challenging. This study aimed to explore the distinctive metabolic changes in plasma samples of AAD patients with hypertension and patients with hypertension only and provide early identification and diagnosis of AAD in patients with hypertension.

**Materials and methods:**

We collected blood samples from 20 patients with type A AAD and hypertension admitted to the emergency department and physically examined other 20 patients with hypertension as controls. The plasma metabolomic profiles of these patients were determined using untargeted metabolomics with ultra-high-performance liquid chromatography-quadrupole time-of-flight mass spectrometry.

**Results:**

A total of 38 metabolites that differed between the AAD and hypertension groups were screened. In the positive ion mode, 12 metabolites were different between the two groups, and in the negative ion mode, 26 metabolites were different. Among the 26 different metabolites detected by the negative ion mode, 21 were significantly upregulated and five were downregulated in patients with AAD compared to patients with hypertension. Moreover, five metabolites were upregulated and seven were significantly downregulated in patients with AAD compared to those with hypertension, as detected by the positive ion mode. The metabolites differentially expressed in AAD were mainly involved in lipid metabolism (fatty acid biosynthesis, biosynthesis of unsaturated fatty acids, and linoleic acid metabolism), carbohydrate metabolism (galactose, fructose, and mannose metabolisms), and membrane transport (ATP-binding cassette transporters). Interestingly, plasma hydrocortisone and dimethylglycine concentrations were significantly increased in patients with type A AAD, with the highest area under the curve value (AUC = 0.9325 or 0.9200, respectively) tested by the receiver operating characteristic curve analysis.

**Conclusion:**

This study provides possible metabolic markers for the early clinical diagnosis of AAD in patients with hypertension.

## Introduction

The normal aortic wall is divided into three layers, the innermost tunica intima, the tunica media, and the tunica adventitia (1). Acute aortic dissection (AAD) is the most devastating aortic pathology caused by the tearing of the tunica intima, which is gradually stripped owing to the impact of bloodstream flow under the action of many pathogenic factors (2). According to the Oxford Vascular Study in Britain, the annual incidence of AAD in developed countries is approximately 6/100,000 (3), and the prevalence of aortic dissection in people over 65 years in the United States is 8%, ranking 13th among the disease killers in the United States (4). In China, diagnostics and living standards have increasingly improved yearly. Due to incomplete statistics, our country has a tendency to experience an increase in the incidence of AAD and show a trend of younger age. Without treatment, the weekly mortality rate for AAD is 37% and increases to 75% in 2 weeks (5). Hypertension is considered one of the most critical risk factors for AAD development and is observed in 65–75% of patients with AAD (6). In China, hypertension diagnosis, treatment, and control have been much lower than in developed countries (7). Despite its importance, reliable estimation of AAD incidence and survival is challenging (8). Therefore, timely screening and diagnosis of AAD in patients with hypertension are of great significance for the prevention, treatment, and prognosis of aortic dissection (9, 10).

Currently, the clinical auxiliary diagnosis of AAD relies mainly on computed tomography angiography (CTA) (11). However, CTA has several limitations. For example, it requires intravenous contrast agents, which should not be used in patients who are allergic to it, and radiation exposure from CTA is not appropriate for particular patients such as pregnant women. Although peripheral blood detection is a rapid and less invasive option, reliable peripheral blood markers for AAD are lacking. The recently identified early peripheral blood biomarkers for AAD [D-dimer, creatine kinase-BB, matrix metalloproteinases (MMPs), and elastin] are not sufficiently specific. Therefore, a sensitive, accurate, non-invasive, and time-sensitive biomarker is needed in clinical practice to facilitate the early detection and intervention of AAD.

Metabolomics has recently emerged as a novel technique to provide comprehensive quantitative measurements of endogenous metabolites within biological systems (12). Metabolomic studies may help better understand the underlying mechanisms and discover new diagnostic markers for AAD. This study aimed to investigate the distinctive metabolic changes in plasma samples from patients with type A AAD and identify sensitive metabolic candidate biomarkers for diagnosing AAD using a metabolomics platform.

## Materials and methods

### Samples from participants

Forty blood samples from 20 patients with type A AAD and hypertension and 20 samples from patients with hypertension only were obtained from the Xiamen Cardiovascular Hospital of Xiamen University from December 2019 to January 2021. The study protocol was approved by the Ethics Committee of Xiamen Cardiovascular Hospital of Xiamen University, and informed consent was obtained from each participant. AAD in patients with hypertension was confirmed by CTA with an accurate and false lumen of the aortic wall. Based on physical examination, hypertensive patients without other cardiovascular diseases were randomly selected from those with a confirmed diagnosis of hypertension in our hospital. Patients with connective tissue disorders or atherosclerotic cardiovascular disease were excluded.

### Metabolite sample preparation

Plasma samples from the 40 patients were slowly thawed at 4°C, and an appropriate amount of the sample was added to a pre-cooled methanol/acetonitrile/water solution (2:2:1, v/v). After eddy mixing, low-temperature ultrasound for 30 min, standing at –20°C for 10 min, and centrifugation at 14000 × g and 4°C for 20 min, the supernatant was vacuum-dried, and 100 μL of acetonitrile-water solution was added during mass spectrometry (acetonitrile:water = 1:1, v/v) for redissolution. After centrifuging the samples at 14000 × g and 4°C for 15 min, the supernatants were ready for analysis (13).

### Ultra-performance liquid chromatography-tandem mass spectrometry analysis

The experimental conditions for chromatography are as follows (14): Samples were separated using an Agilent 1290 Infinity LC ultra-performance liquid chromatography (UHPLC) column. The flow rate was 0.5 mL/min, and the injection volume was 2 μL. Mobile phase composition: A was water with 25 mM ammonium acetate and 25 mM ammonia and B was acetonitrile. The gradient elution procedure was as follows: 0–0.5 min, 95% B; 0.5–7 min, B changed linearly from 95 to 65%; 7–8 min, B changed linearly from 65 to 40%; 8–9 min, B remained at 40%; 9–9.1 min, B changed linearly from 40 to 95%; 9.1–12 min, B was maintained at 95%. The samples were placed in an automatic sampler at 4°C throughout the analysis. To avoid the influence of the fluctuation of the instrument detection signal, continuous analysis of the samples was carried out in a random sequence. The quality control (QC) samples were inserted into the sample queue to monitor and evaluate the stability and reliability of the experimental data.

The ESI source conditions were set as follows: Ion Source Gas1 (Gas1), 60; Ion Source Gas2 (Gas2), 60; curtain gas (CUR), 30; source temperature, 600°C; and ion spray voltage floating, ± 5500 V. In MS acquisition, the instrument was set to acquire over the mass-to-charge ratio (m/z) range of 60–1000 Da, and the accumulation time for the TOF MS scan was set at 0.20 s/spectra. In the auto MS/MS acquisition, the instrument was set to acquire over the m/z range of 25–1000 Da, and the accumulation time for the production scan was set at 0.05 s/spectra. The production scan was acquired using information-dependent acquisition with a high-sensitivity mode. The parameters were set as follows: the collision energy was fixed at 35 V with ± 15 eV; de-clustering potential, at 60 V (+), and –60V (–), excluding isotopes within 4 Da; and candidate ions to monitor per cycle, 10.

### Quality controls

#### Comparison of total ion flow diagrams of quality control samples

The total ion chromatograms of the QC samples were superimposed and compared. The experimental results showed that each chromatographic peak’s response strength and retention time overlapped, indicating that the variation caused by instrument error was slight during the entire experimental process.

#### Principal component analysis of the total samples

Principal component analysis (PCA) was performed on the peaks extracted from all experimental and QC samples. The results showed that QC samples in the positive and negative ion modes were closely clustered together, indicating good reproducibility of the experiment.

### Data processing

The raw MS data (wiff.scan files) were converted to MzXML files using ProteoWizard MS Convert before importing them into the freely available XCMS software. For peak picking, the following parameters were used: center-wave m/z = 25 ppm, peak width = c (10, 60), and prefilter = c (10, 100). For the peak grouping, bw = 5, mzwid = 0.025, and minfrac = 0.5. Collection of Algorithms of MEtabolite pRofile Annotation (CAMERA) was used to annotate isotopes and adducts. Only variables with >50% of the non-zero measurement values in the extracted ion features were kept in at least one group. Compound identification of metabolites was performed by comparing the accuracy of m/z values (<25 ppm) and MS/MS spectra with an in-house database established with authentic standards.

### Statistical analyses

After normalization to the total peak intensity, the processed data were analyzed using the R package (ropes), where they were subjected to multivariate data analysis, including Pareto-scaled PCA and orthogonal partial least-squares discriminant analysis (OPLS-DA). The 7-fold cross-validation and response permutation testing were performed to evaluate the robustness of the model. The variable importance in the projection (VIP) value of each variable in the OPLS-DA model was calculated to indicate its contribution to the classification. Metabolites with VIP values >1 were further subjected to Student’s *t*-test at the univariate level to measure the significance of each metabolite. Statistical significance was set at *p* < 0.05.

## Results

### General data of participants

Forty participants, including 20 patients with AAD plus hypertension and 20 with hypertension only from the Xiamen Cardiovascular Hospital of Xiamen University, were recruited. The detailed data of the two groups are summarized in Table 1. There were no significant differences in age, sex, height, weight, or body mass index between the AAD and hypertension groups. Urea, blood glucose, creatinine, and uric acid values did not differ between the AAD and hypertension groups. Both groups were diagnosed with hypertension: systolic blood pressure (148.60 ± 17.25 in the hypertension group vs. 147.30 ± 28.32 in the AAD group, *P* = 0.8618) and diastolic pressure (91.80 ± 9.61 in the hypertension group vs. 93.15 ± 14.17 in the AAD group, *P* = 0.7263). Moreover, the D-dimer (10.84 ± 10.94 mg/L) and hs-CRP (35.54 ± 53.40 mg/L) of the AAD group were higher than the standard value range (<0.55 mg/L or <3 mg/L), but these values were not detected in the hypertension group.

**TABLE 1:** The clinical information of AAD patients and hypertension patients.

Items	Hypertension (*n* = 20)	Acute aortic dissection (*n* = 20)	*P*-value
Age (years)	56.35 ± 15.30	58.10 ± 15.30	0.6472
Gender ratio (M/F)	12:8	10:10	–
Height (cm)	162.20 ± 8.76	160.70 ± 8.16	0.5876
Weight (kg)	58.85 ± 10.40	59.50 ± 10.11	0.8423
BMI (kg/m^2^)	22.25 ± 2.63	22.97 ± 3.10	0.4317
Systolic blood pressure (mmHg)	148.60 ± 17.25	147.30 ± 28.32	0.8618
Diastolic pressure (mmHg)	91.80 ± 9.61	93.15 ± 14.17	0.7263
D-dimer (mg/L)	Not detected	10.84 ± 10.94	–
Hs-CRP (mg/L)	Not detected	35.54 ± 53.40	–
Urea (mmol/L)	6.84 ± 4.78	7.48 ± 3.55	0.6362
Blood glucose (mmol/L)	7.47 ± 1.63	8.62 ± 2.75	0.1167
Creatinine (mmol/L)	82.68 ± 63.07.6	83.53 ± 29.96	0.9566
Uric acid (mmol/L)	379.90 ± 126.76	329.34 ± 141.45	0.2413

### Statistical analysis of samples and distributions of different metabolites

**FIGURE 1:**
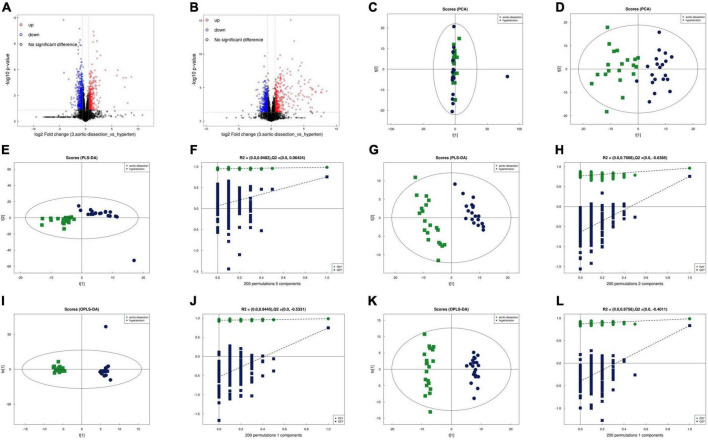
Separation and classification of metabolic profiles in plasma from the AAD and hypertension groups. Volcano plot of positive **(A)** and negative **(B)** ion mode results. PCA score plots of the AAD and hypertension groups in the positive **(C)** and negative **(D)** ion modes. PLS-DA score plots of AAD and hypertension groups in the positive **(E)** and negative **(F)** mode and the cumulative fitness (R^2^ value) and prediction power (Q^2^ value) of the PLS-DA analysis in the positive **(G)** and negative **(H)** ion mode. OPLS-DA score plots of AAD and hypertension groups in the positive **(I)** and negative **(J)** ion mode and the cumulative fitness (R^2^ value) and prediction power (Q^2^ value) of the OPLS-DA analysis in the positive **(K)** and negative ion modes **(L)**. The t [1] and t [2] values in the figures represent the scores of each sample in principal components 1 and 2, respectively. Each dot on the plot represents a sample in the corresponding group. PCA: principal component analysis; PLS-DA: orthogonal partial least square discriminant analysis; PLS-DA: a partial least square discriminant analysis.

The volcano maps with *p* < 0.05 and log_2_FC > 0.5 are shown as positive (Figure 1A) and negative (Figure 1B) ion modes, respectively. Before analyzing the differentially expressed metabolites, we validated the algorithm model used in the analysis using PCA, partial least squares discrimination analysis (PLS-DA), and OPLS-DA. PCA is an unsupervised data analysis method that re-linearly combines all identified metabolites to form a new set of comprehensive variables and selects some of them according to the analyzed problem. The distribution of metabolic profiles for the samples in the PCA was exhibited in ESI positive (Figure 1C) and negative (Figure 1D) ion modes. PCA results showed that the two groups could not be effectively separated. In the subsequent analysis, the two methods, PLA-DA and OPLS-DA were applied to eliminate information and perform orthogonal signal correction to further process the above results. PLS-DA is supervised statistical discriminant analysis. OPLS-DA is a modified analytic method for PLS-DA that can filter out noises irrelevant to classification information and improve the analytical ability and effectiveness of the model. The PLS-DA (Figure 1E, positive ion mode; Figure 1G, negative ion mode) and OPLS-DA (Figure 1I, positive ion mode; Figure 1K, negative ion mode) score plots in both ESI positive and negative modes indicated that the cluster of patients with AAD was well separated from those with hypertension, suggesting that the overall metabolic pattern was altered in the plasma of both patients with AAD plus hypertension and hypertension only. Specifically, the explanation rates for the Y variable (R2Y) and prediction ability (Q2) were 0.9482 and 0.06424 in the positive ion mode (Figure 1F) and 0.7668 and –0.6385 in the negative ion mode (Figure 1H) in the PLS-DA model, respectively. In the OPLS-DA, R2Y and Q2 were 0.9445 and –0.5331 in the positive ion mode (Figure 1J) and 0.8755 and –0.4011 in the negative ion mode (Figure 1L), respectively, which confirmed the stability and reliability of the OPLS-DA mode.

**FIGURE 2:**
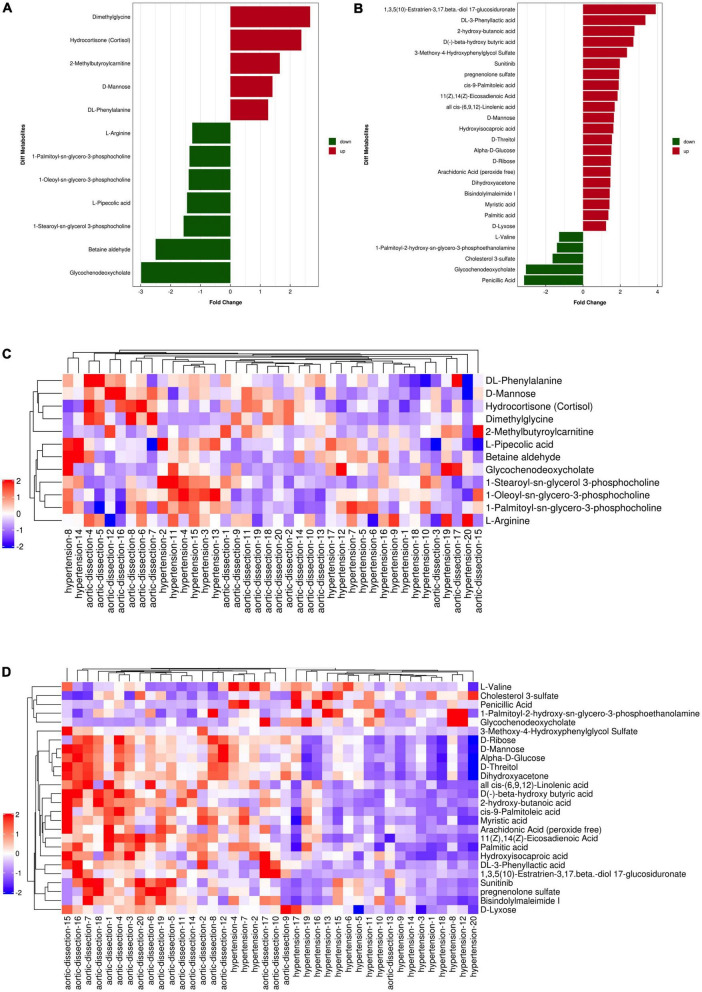
Fold change analysis and heat map of the differential metabolites between hypertension and AAD groups in ESI positive and negative mode. **(A)** Fold change of five up-regulated metabolites (red bars) and seven down-regulated metabolites (green bars) in ESI positive ion mode. **(B)** Fold change of 21 up-regulated metabolites (red bars) and five down-regulated metabolites (green bars) in ESI negative mode. Heat map of the differential metabolites between hypertension and AAD groups in ESI positive **(C)** and negative **(D)** ion mode (red represents the metabolites in high abundance, blue represents the metabolites in low abundance).

A total of 9902 and 9337 m/z features were detected in the positive and negative ion modes, respectively, from the plasma samples of the two groups. In the positive mode, 12 m/z features were significantly different between patients with AAD and hypertension (*P* < 0.05); 5 m/z features showed a fold change (FC) > 1, and 7 m/z features showed an FC < 1 between patients with AAD and hypertension (*P* < 0.05) (Figures 2A,C and Table 2). In the negative mode, 26 m/z features were significantly different between patients with AAD and hypertension (*P* < 0.05); 21 m/z features had an FC > 1.5, and 5 m/z features had an FC < 1 (Figures 2B,D and Table 3).

**TABLE 2:** Statistical analysis of different metabolites in positive ion patterns.

Name	VIP	Fold change	*P*-value	m/z	rt(s)
Hydrocortisone (Cortisol)	2.540181454	2.377060532	2.54044E-07	363.21489	45.067
Dimethylglycine	1.238043185	2.669990192	1.84116E-05	104.06966	290.683
1-Palmitoyl-sn-glycero-3-phosphocholine	16.31693226	0.731060704	9.82702E-05	496.33927	189.075
L-Pipecolic acid	3.108120087	0.689489648	0.001123665	171.11275	258.895
D-Mannose	2.074941992	1.40761344	0.001905338	198.09617	291.5135
Glycochenodeoxycholate	1.283728085	0.33516846	0.002624755	432.30876	203.9575
Betaine aldehyde	4.134241653	0.400500219	0.002885916	162.11226	310.12
1-Oleoyl-sn-glycero-3-phosphocholine	6.57882624	0.717045917	0.003526007	522.35398	154.556
1-Stearoyl-sn-glycerol 3-phosphocholine	2.22919433	0.640546032	0.010722346	524.3687	84.6435
DL-Phenylalanine	1.766904818	1.263807289	0.017858202	207.11233	225.446
2-Methylbutyroylcarnitine	3.235520714	1.654094994	0.036214924	246.16943	234.765
L-Arginine	3.384491698	0.785754592	0.049182685	175.11869	578.0455

**TABLE 3:** Statistical analysis of different metabolites in negative ion patterns.

Name	VIP	Fold change	*P*-value	m/z	rt(s)
L-Glutamate	1.75684417	0.425108584	9.73216E-06	146.04467	394.25
11(Z),14(Z)-Eicosadienoic Acid	3.256119257	0.580386243	0.00121708	307.2624	45.183
Arachidonic Acid (peroxide free)	8.785229564	0.491955015	0.00146021	303.23177	45.179
Myristic acid	6.179872893	0.571095792	0.001648591	227.20056	48.539
DL-lactate	12.94141146	0.400099692	0.002179355	89.02434	266.916
16-Hydroxypalmitic acid	1.382002127	0.664183457	0.00232748	271.22585	74.643
1-Palmitoyl-2-hydroxy-sn-glycero-3-phosphoethanolamine	2.068817045	0.637222361	0.00307604	452.27658	194.745
1-Oleoyl-L-.alpha.-lysophosphatidic acid	1.056618982	0.267034475	0.003457425	457.23309	239.6715
2-hydroxy-butanoic acid	1.757680909	0.483444895	0.003683016	103.03934	193.2195
Palmitic acid	17.13490388	0.738098272	0.004073127	255.23278	47.299
all cis-(6,9,12)-Linolenic acid	8.921459175	0.522121985	0.004282796	277.21612	46.843
Nname,cis-9,10-Epoxystearic acid	1.841099778	0.712405004	0.004650072	297.24161	50.606
Pentadecanoic Acid	1.08088633	0.704023246	0.005292252	241.21579	47.915
L-Valine	2.489268942	0.59057917	0.005351996	116.07095	301.4365
Taurine	2.86744555	0.681677383	0.007222477	124.00697	289.48
Hypoxanthine	2.988244266	0.46944798	0.009166969	135.03041	164.6565
Glycerol	1.422678731	0.389901064	0.01029718	92.04968	80.8635
D-Arabinono-1,4-lactone	1.416010553	1.373664747	0.011288645	147.02873	150.67
1-Palmitoyl Lysophosphatidic Acid	1.143848211	0.207112312	0.011523676	409.23304	245.3135
L-Proline	1.24529976	0.708887843	0.014415796	114.05519	308.705
Cyanuric acid	1.13649118	1.310366919	0.016014213	129.01818	195.272
3-Methoxy-4-Hydroxyphenylglycol Sulfate	1.627694095	0.485754533	0.016688262	263.02162	44.291
Formylanthranilic acid	2.024688357	0.540934721	0.025152884	164.034	95.876
cis-9-Palmitoleic acid	2.59147119	0.685273976	0.027505613	253.21627	34.451
L-Gulonic gamma-lactone	1.138848495	1.267933006	0.029405392	237.05989	125.187
D(-)-beta-hydroxy butyric acid	1.004123231	0.594452067	0.033847279	103.03919	213.233
Valsartan	1.59310605	0.091676265	0.037294106	434.2198	98.984
Methylmalonic acid	1.896513445	1.216446702	0.044149286	117.0183	127.685

### Identification of different metabolites between the acute aortic dissection and hypertension groups

According to the normality of the data and the homogeneity of variance, we selected the univariate ANOVA test and Kruskal–Wallis test (*p* < 0.05) to measure the differences in metabolites between the two groups. The fold change of significantly differential metabolites in AAD versus hypertension in positive and negative ion modes is presented in Figures 2A,B and, respectively. Specifically, in the positive ion mode, dimethylglycine and hydrocortisone were more than 2-fold upregulated in the AAD group, and glycochenodeoxycholate and betaine aldehyde showed more than 2-fold downregulation compared with the hypertension group. In the negative ion mode, 1,3,5(10)-Estratrien-3,17 beta-diol 17-glucosiduronate, DL-3-phenylacetic acid, 2-hydroxy-butanoic acid, D(-)-beta-hydroxybutyric acid, and 3-methoxy-4-hydroxyphenylglycol sulfate were upregulated by more than 2-fold, and penicillic acid and glycochenodeoxycholate were downregulated by more than 2-fold in the AAD group compared with the hypertension group. Moreover, a hierarchical clustering heatmap was used to view the data more intuitively (Figures 2C,D). Cluster analysis of all the samples revealed that the AAD and hypertension groups clustered well. The heatmap suggests that the levels of metabolic biomarkers in the AAD group differed from those in the hypertension group.

### Potential signaling pathways

**FIGURE 3:**
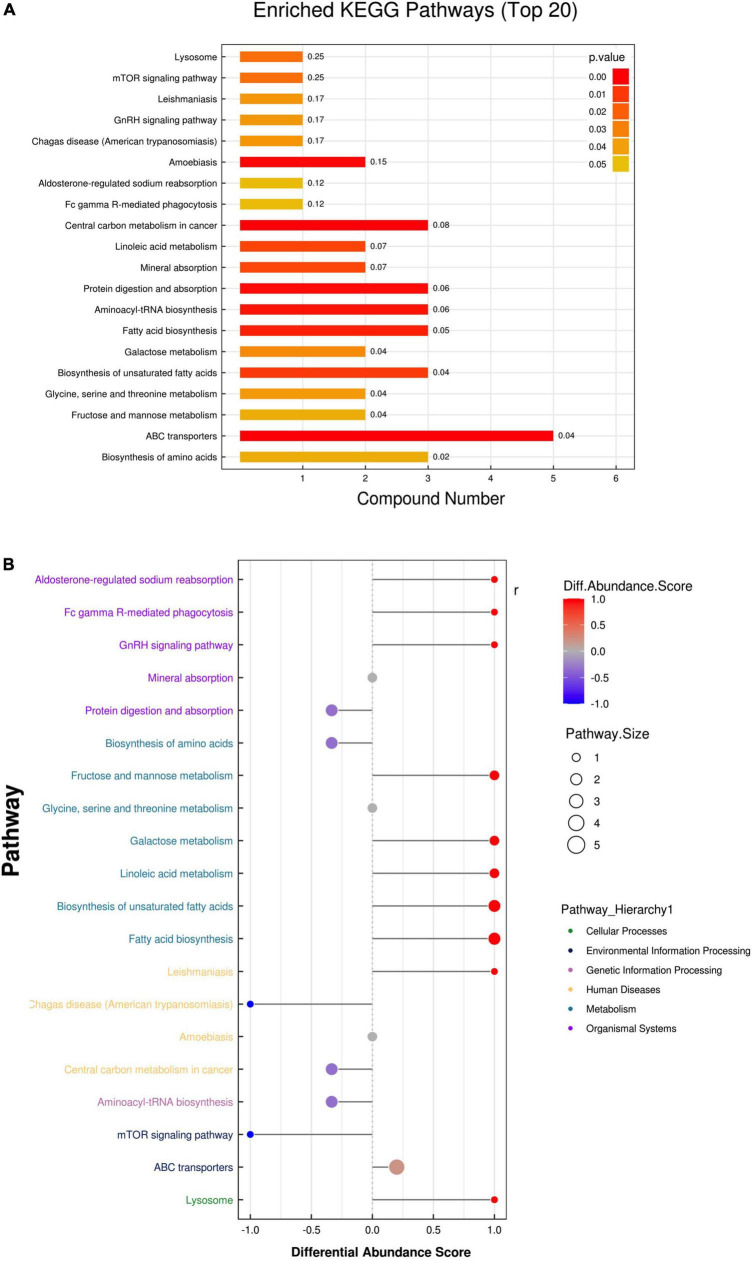
Enrichment analysis of the metabolic pathway of the two groups. **(A)** The enrichment analysis of the Kyoto Encyclopedia of Genes and Genomes (KEGG) showed the overview pathway of different endogenous metabolites between HP and AD groups. **(B)** Differential abundance score map for all differential metabolic pathways analyzed by Pathway Hierarchy.

To reveal the pathways of metabolites and their metabolic processes, MetaboAnalyst 4.0^1^ was performed for pathway enrichment analysis (Figure 3). According to the KEGG and hierarchy pathway results, the major intervened pathways involved were ABC transporters, biosynthesis of amino acids, fructose and mannose metabolism, unsaturated fatty acids and fatty acid biosynthesis, and aminoacyl-tRNA biosynthesis (Figures 3A,B). Combined with the classification of differential metabolites, lipid metabolism (fatty acid biosynthesis, biosynthesis of unsaturated fatty acids, and linoleic acid metabolism), carbohydrate metabolism (galactose, fructose, and mannose metabolism), and membrane transport (ABC transporters) were the central pathways in the AAD group. In addition, two classical pathways, the mTOR signaling pathway, and aldosterone-regulated sodium reabsorption are worthy of attention. These results indicate that AAD may be affected by fatty acids, carbohydrate, and lipid metabolic pathways.

### Diagnostic accuracy of metabolites

**FIGURE 4:**
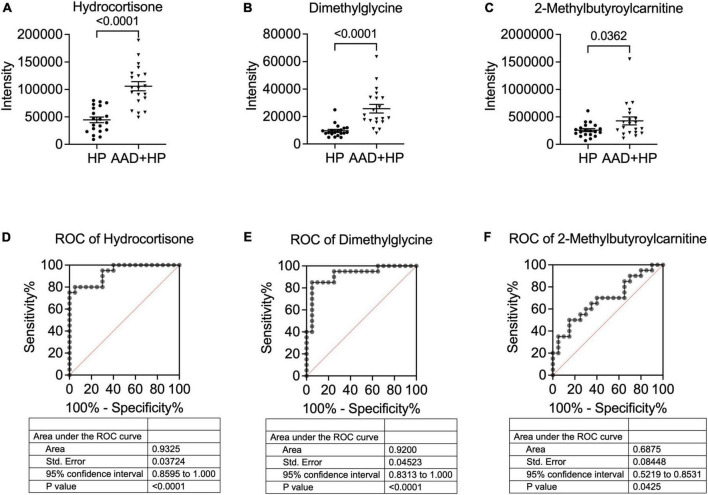
Concentration and ROC analysis of candidate metabolites in the two groups. Comparison of hydrocortisone blood levels. **(A)** dimethylglycine **(B)** and 2-methylbutyroylcarnitine **(C)** in patients with hypertension and AAD + hypertension. The ROC analysis showed that hydrocortisone **(D)** exhibited the highest AUC value, followed by dimethylglycine **(E)** and 2-Methylbutyroylcarnitine **(F)**. AUC, Area under the curve; ROC, receiver operating characteristic.

Among the 26 differentially expressed metabolites, we found that three metabolites were upregulated with *p* < 0.05 and more than 1.5-fold change (Figures 4A–C). We then tested their ability to differentiate AAD from hypertension using the receiver operating characteristic (ROC) curve analysis. According to the ROC analysis, the diagnostic power of hydrocortisone was the highest among the three candidate metabolites [AUC: 0.9325; 95% confidence interval (CI): 0.8595–1.000] (Figure 4D), followed by that of dimethylglycine (AUC: 0.9200; 95%CI: 0.8313–1.000) (Figure 4E) and 2-methylbutyroylcarnitine (AUC: 0.6875; 95%CI: 0.5219–0.8531) (Figure 4F), indicating that hydrocortisone and dimethylglycine may be potential metabolic markers for diagnosing AAD in hypertension.

## Discussion

Acute aortic dissection (AAD), one of the most dangerous and fatal cardiovascular diseases (15), has an acute onset in severe conditions; the early mortality rate of untreated AAD is approximately 1–2% per hour (16). Dissection of the aortic wall can be caused by several factors such as genetic diseases and aortic valve devaluation, but the most common is hypertension (8, 16). In China, many people have hypertension. However, many of them are not well controlled because of the irregular use of antihypertensive drugs, which may lead to aortic dissection. Therefore, early screening and precise diagnosis of aortic dissection in patients with hypertension are critical for the treatment and prognosis of this disease.

Metabolomics is an extension of transcriptomics and proteomics that can directly and accurately reflect the physiological state of an organism (17). The differences in metabolic levels may be biomarkers for diagnosing diseases and exploring their severity, studying the biological and pathological processes of different conditions, and revealing their mechanisms and regulatory pathways. Although patients with AAD have low rates of previous vascular disease, including ischemic heart disease, diabetes mellitus, and peripheral arterial disease, previous observations have confirmed that hypertension is strongly associated with AAD (18, 19). Uncontrolled hypertension remains the most significant risk factor for AAD (3). Therefore, exploring the markers of AAD in patients with hypertension is crucial for the early diagnosis of aortic dissection. This study aimed to investigate the differential metabolites between patients with AAD (and hypertension) and patients with hypertension. We enrolled 20 patients with AAD who were also diagnosed with hypertension and 20 patients with hypertension as controls. After metabolomics and further analysis, 12 and 26 metabolites were found to have a significant difference in the positive and negative ion modes, respectively. Five metabolites were upregulated and seven were downregulated in the positive ion mode, and five were upregulated, and 21 were downregulated in the negative ion mode. In the enrichment pathway analysis, monosaccharides, fatty acids, and metabolic products of amino acids were different between patients with AAD and those with hypertension. Specifically, dimethylglycine, hydrocortisone,1,3,5(10)-Estratrien-3,17 beta-diol 18-glucosiduronate, DL-3-phenylacetic acid, 2-hydroxy-butanoic acid, D(-)-beta-hydroxybutyric acid, and 3-methoxy-4-hydroxyphenylglycol sulfate were up-regulated over 2-fold.

Many previous studies in the past decade have explored biomarkers for AAD using metabolomics. Cui et al. (20) demonstrated that plasma succinate concentrations distinguished patients with AAD from both healthy controls and patients with acute myocardial infarction or pulmonary embolism. Zeng et al. (2) found that trimethylamine N-oxide (TMAO) levels were increased in patients with AAD compared with those in healthy controls, which was positively correlated with C-reactive protein, interleukin-6, and D-dimer levels. In another previous study, the authors found a total of 236 differentially expressed metabolites in patients with AD compared with healthy participants, of which 142 were significantly upregulated and 64 significantly downregulated, and 165 differentially expressed metabolites in patients with AD compared with patients with hypertension, of which 109 were significantly downregulated and 56 were upregulated. They speculated that two of these metabolites [tetranor-PGE1 and 13, 14-dihydro-19 (R)-hydroxyPGE1] might serve as potential plasma biomarkers for AD (21). Zhou et al. indicated that LPCs and sphingolipids are significantly altered in patients with AAD, particularly in Stanford type A AAD, where several sphingolipids, such as sphingosine, phytosphingosine, and ceramide were significantly decreased (22). In a study of thoracic aortic dissection (TAD), the authors performed untargeted metabolomics to determine plasma metabolite concentrations in an aortic disease cohort. They found that C18-ceramide was significantly differentiated in patients with TAD, but not in patients with thoracic aortic aneurysms, which was confirmed by subsequent quantitative analysis of C18-ceramide in a validation cohort. The ceramide synthesis pathway was inhibited by myriocin-alleviated BAPN-induced aortic inflammation and dissection in mice. *In vitro* studies found that exogenous C18-ceramide promoted macrophage inflammation and matrix metalloproteinase (MMP) expression through the NLRP3-caspase 1 pathway (23). However, in our metabolomics results, the plasma levels of succinate, TMAO, tetranor-PGE1, 13, 14-dihydro-19 (R)-hydroxyPGE1, LPCs, and sphingolipids did not differ between patients with AAD and those with hypertension, indicating that these metabolites might not be critical markers for diagnosing AAD patients with hypertension.

In this study, fatty acid biosynthesis was identified as a critical pathway for AAD. Fatty acid synthesis occurs in both the cytosol and mitochondria of most organs, although one or the other predominates in a given organ. The spectrum of fatty acids synthesized by the whole aorta or its constituent subfractions is not notably different from that of other organs (24). Husi et al. found decreased glycolysis and fatty acid metabolism and a pronounced increase in vascularization, oxidative stress-response proteins, apoptosis modulators, and molecules involved in hypertrophy and hypertension (25). Carbohydrate metabolism (galactose, fructose, and mannose metabolisms) is another key pathway in the differentiation of patients with AAD and hypertension. A previous study showed that carbohydrate metabolism disturbances affected the wall of the aorta and the development of alimentary lipidosis in rabbits (26). The state of carbohydrate metabolism and the microcirculation in ischemic muscles of the extremities may regulate the embolism of the aorta (27). The increased deposition of cholesterol in the arterial intima may result in vascular dysfunction, which is a key event in the pathogenesis of atherosclerosis and aortic dissection. The ABC transporter family plays an important role in the balance between lipid accumulation and lipid removal from cells (28). Our results also demonstrate the critical role of ABC transporters in the early stages of AAD. Another important finding of the present study is that hydrocortisone and dimethylglycine showed the strongest relationship with type A AAD. Interestingly, dimethylglycine is also elevated in the blood of children with congenital heart defects and their mothers (29). Moreover, dimethylglycine is elevated in plasma and associated with and improved risk prediction of mortality in patients with suspected or verified coronary heart disease (30). Hydrocortisone is an essential regulator of aortic dysfunction and other cardiovascular diseases. The inhibition of vascular PGI2 by hydrocortisone has implications in the pathogenesis of steroid-related hypertension and atherosclerosis and the anti-inflammatory effect of steroids (31). Hydrocortisone also increased heart rate and blood pressure and reduced cardiovagal baroreflex sensitivity and heart rate variability in young men (32). Pretreatment with a stress dose of hydrocortisone but not a higher pharmacologic dose maintained a significantly higher approximate entropy after endotoxin exposure compared to a placebo (33). Our group and other results indicated that dimethylglycine and hydrocortisone play an important role in regulating heart or cardiovascular disorders.

## Conclusion

In conclusion, our study provides new evidence that 38 metabolites differ between the type A AAD and hypertension groups. These metabolites are involved in lipid metabolism (fatty acid biosynthesis, biosynthesis of unsaturated fatty acids, and linoleic acid metabolism), carbohydrate metabolism (galactose, fructose, and mannose metabolism), and membrane transport (ABC transporters), thus regulating AAD progression. Hydrocortisone and dimethylglycine concentrations were significantly increased in type A AAD patients, with the highest area under the curve value, and may serve as potential biomarkers for AAD. These results are of great importance in the exploration of biomarkers and regulators for the early diagnosis and treatment of AAD; however, further *in vitro* and *in vivo* studies are required to evaluate the potential role of hydrocortisone and dimethylglycine and their related pathways in AAD.

## Data availability statement

The raw data supporting the conclusions of this article will be made available by the authors, without undue reservation.

## Ethics statement

The studies involving human participants were reviewed and approved by the Ethics Committee of Xiamen Cardiovascular Hospital of Xiamen University. The patients/participants provided their written informed consent to participate in this study.

## Author contributions

JZ and H-FQ designed the experiments. X-BH and YH conducted the experiments. X-BH and GL prepared the manuscript. JZ, H-FQ, and X-JW supervised the study, revised the manuscript, and provided funding. E-RN and M-CY validated the results. All authors contributed to writing and editing the manuscript and read and approved the final manuscript.

## Footnotes

1
https://www.metaboanalyst.ca/

## Abbreviations

AAD: acute aortic dissection
ROC: receiver operating characteristic
AUC: area under the curve
CTA: computed tomography angiography
UHPLC: ultra-performance liquid chromatography
PCA: principal component analysis
PLS-DA: partial least squares discrimination analysis
OPLS-DA: orthogonal partial least squares discriminant analysis
TMAO: trimethylamine N-oxide
MMP: matrix metalloproteinase
QC: quality control.
